# Street centrality and vitality of a healthy catering industry: A case study of Jinan, China

**DOI:** 10.3389/fpubh.2022.1032668

**Published:** 2022-11-24

**Authors:** Yanbin Chen, Guanwen Yin, Yiming Hou

**Affiliations:** College of Geography and Environment, Shandong Normal University, Jinan, Shandong, China

**Keywords:** healthy catering industry, street centrality, vitality, geographical detectors, multiscale geographically weighted regression

## Abstract

In the context of an overall improvement in the national economy, residents' demand for nutrition and health has been increasing. An industry that provides healthy eating plays an increasingly important role in urban development. Few studies, however, have focused on the relationship between the urban road network structure and the vitality of the healthy catering industry (VHCI). Based on social media data and urban traffic network data, in this study, we explored the impact mechanism of street centrality on the VHCI through a case study of Jinan, China, using geographic detectors and multiscale geographically weighted regression (MGWR) methods. The results show the following: First, the vitality of the catering industry in the main urban area of Jinan has multicore spatial distribution characteristics, and the high-value areas of the vitality of the catering industry are highly matched with the main business districts in the city in space. Second, we found clear spatial differences in street centrality between the walking and driving modes. With an increase in the search radius, the trend of high-value areas closely gathering in the urban center became clearer. The distribution of betweenness was observed from sheet to grid, and the multicenter structure of straightness was more prominent. Third, differences in the residents' perception of the road network structure caused by different travel modes affected their choice of dining places. In the driving mode, betweenness and straightness had a greater impact on the vitality of the catering industry, and the effect of closeness in the walking mode was more obvious. Fourth, the influence of street centrality on the vitality of the healthy catering industry had obvious spatial heterogeneity. In the walking mode, the spatial heterogeneity of straightness was the strongest, followed by betweenness, and closeness was the weakest; in the driving mode, the spatial heterogeneity of closeness was the strongest, followed by straightness, and betweenness was the weakest. From the perspective of residents' travel, the results of this study revealed the influence mechanism of urban road network characteristics on the VHCI. This information can aid planning for urban space optimization and improve residential living quality.

## Introduction

The concept of “vitality” originated from biology and ecology, which originally meant vigorous vitality. Jacobs first proposed the concept of “urban vitality” and believed that the process of interweaving people's activities and living places constituted the diversity of urban life and enabled the city to obtain vitality (1). Gehl noted that urban vitality comes from the people and the activities of the people in the urban space (2). Based on previous studies, Montgomery proposed that the components of urban vitality include “activity,” “transactions,” and “diversity” (3). With the development of society and the economy, this theory of “vitality” has been introduced gradually into various fields of urban life, such as industrial development (4, 5), public health (6), and criminology (7). Today, the consumer economy has shifted gradually from products to services, and the significant demand generated by consumers has become an important force driving economic and social development (8). As an important part of the urban service industry, the catering industry has shown strong development potential and has become a dynamic source of urban vitality (9). In recent years, the emphasis on high-quality life has led people to pursue healthy green diets (10). Nutrition, health, and wellness have become the major trends in the development of the catering industry (11). Therefore, in the context of the construction of “Healthy China” (China's development strategy to improve national health policies and provide people with all-round and full-cycle health services), researching restaurants that provide healthy diets from the perspective of urban vitality not only is conducive to promoting the green development of the catering industry but also can satisfy people's ever-growing needs for a better life.

The study of urban catering industry has always been one of the focuses of urban geography and urban planning. According to the gravitational model, traditional research has analyzed the location characteristics of the urban catering industry, emphasizing the role of centrality and distance (12, 13). With the improvement of the convenience of spatial data acquisition, research on the urban catering industry has continued to deepen. From the perspective of research methods, the existing research mainly obtains spatial location data of catering outlets through network technology, by combining ArcGIS software and measurement models to analyze the spatial layout characteristics and influencing factors of the catering industry (14–16). For example, Girish et al. used a gravity model to assess the factors influencing restaurant development (17); Lu et al. (18) analyzed the spatial distribution characteristics of urban economic and geographical elements using methods such as location entropy and nearest neighbor index; and Zhang et al. (19) quantitatively analyzed the hierarchical hotspots and spatiotemporal structures of catering services in Chongqing, China, based on kernel density estimation (KDE) method. From these research results, most scholars believe that the distribution of urban catering merchants generally presents a circular divergence law from the urban central business district to the surrounding areas. The interior of each area appears to form a combination of clustered agglomeration and banded distribution (20, 21). The rise of big data has provided new opportunities for the research of the urban catering industry (22, 23). In addition to the research on the geographical distribution and influencing factors of the catering industry, this research has gradually extended to include the online word-of-mouth effect of the catering industry (24, 25), local population catering preferences (26), catering consumption behavior (27), and other aspects. For example, Xu et al. established a word-of-mouth evaluation index system based on network big data to study the spatial pattern of the catering industry in Nanjing, China (28), and Tian et al. revealed the interaction between residents' dining activities and urban space through the mining and analysis of social media data (29). Overall, the current research results on the urban catering industry have been relatively rich, but few studies have examined healthy catering. In particular, the research on the urban healthy catering industry from the perspective of vitality needs to be further strengthened.

Transportation is an important factor affecting the development of the catering industry. The synergistic relationship between urban transportation and the catering industry has always been the focus of scholars (30). In terms of the relationship between transportation and catering, typical research has included the following: Li et al. (31) analyzed the layout of the catering industry in Lhasa based on ArcGIS software and found that catering outlets tend to be concentrated in areas with high traffic integration; and Njomo analyzed the spatial distribution of catering outlets in Nairobi County, Kenya, and found that a convenient road network could bring more customers to restaurants and increase profits (32). In recent years, with the introduction of complexity science and the advancement of digital information technology, the network characteristics of transportation elements have become a consensus. Street centrality is an effective means to interpret the spatial characteristics of the transportation network (33)—that is, the better the centrality, the better the location conditions. Therefore, the relationship between the distribution of urban social activities and economic activities and street centrality has received considerable attention. The multiple centrality assessment (MCA) model is an important method used for centrality evaluation. This method introduces the real distance of the road network into the road network model, and it reflects the node centrality by measuring the importance of the node in the road network, which has strong practical significance (34). At present, the MCA model has been used widely in many aspects of urban life, such as land space optimization (35, 36), traffic orientation detection (37), and traffic flow analysis (38). In addition, many studies have proved that street centrality has an important influence on the creation of urban vitality. For example, Rui et al. used the MCA model to study the relationship between street centrality and land use, and they found that different street centrality indicators could reflect different human activity patterns (39). Kang studied the impact of urban road networks on residents' travel and found that the characteristics of road network structures that are attractive to residents' walking vary with travel scales (40). Sarkar et al. studied the comprehensive impact of urban greening and road network intermediary on residents' activities, and they found that the higher the road network intermediary, the stronger the willingness of residents to walk (41). These studies have explored the connection between urban vitality and street centrality; however, some limitations persist. First, research is lacking on the relationship between urban street centrality and the vitality of the catering industry; second, residents' perception of the road network structure is different because of different travel scales, and therefore, the role of the street centrality index in different travel modes of residents should be further explored; and, third, most of the studies have conducted global modeling of the relationship between street centrality and economic activities but have lacked local modeling analysis. As a result, the local characteristics of the relationship between street centrality and residents' activities has been ignored.

According to this analysis, this study promotes the development of the existing literature in the following three ways: first, we focused on restaurants that provide healthy meals and explore their vitality; second, based on the MCA model, we calculated the street centrality of residents' travel under different traffic modes (walking and driving) and compared the differences in the impact of road network structure on the VHCI; and third, using a MGWR model, we revealed that the spatial heterogeneity of street centrality affects the VHCI. Specifically, we selected Jinan, China, as the research area. We selected restaurants that meet the standards of healthy eating and used the number of restaurant comments on Dianping.com to characterize their vitality. Then we analyzed the spatial characteristics of the vitality of the catering industry and street centrality. Finally, using Spearman's correlation coefficient, geographic detectors, and the MGWR model, we analyzed the influence mechanism of street centrality on the vitality of the catering industry under different travel modes. This study deepens our understanding of the relationship between road network structure and urban economic activities and provides policy guidance for the optimization of urban dining space and improvements in residential quality of life.

## Materials and methods

### Study area

**Figure 1:**
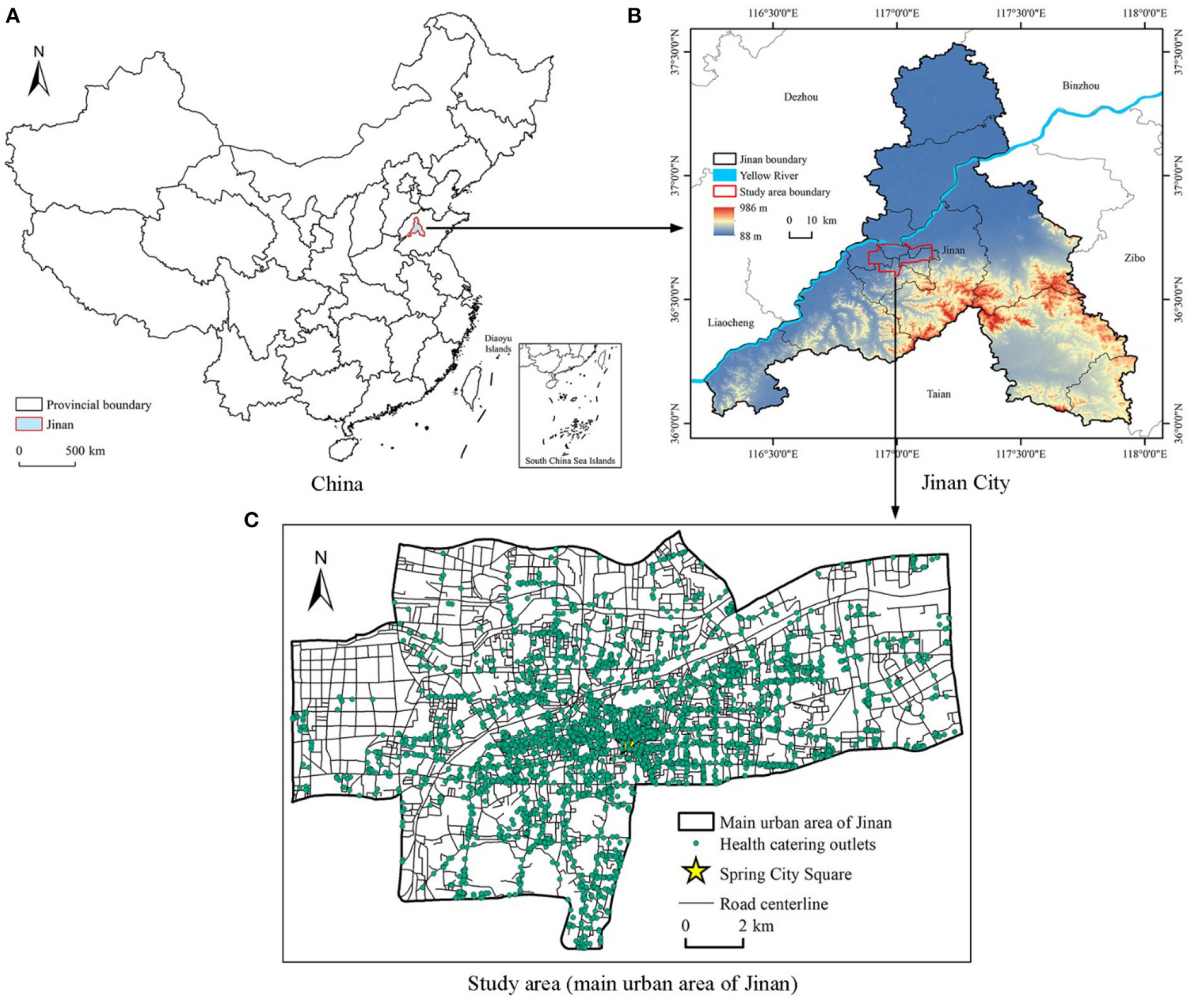
Study area and geographical location of Jinan. **(A)** China. **(B)** Jinan City. **(C)** The main urban area of Jinan.

Jinan is located in the central and western parts of Shandong Province, China, with Mount Tai in the south and the Yellow River in the north. The terrain is high in the south and low in the north. Restricted by geographical conditions, the old city of Jinan expands in an east–west direction, forming a long and narrow urban space from east to west. As the provincial capital of Shandong Province, Jinan is the political, economic, and cultural center of the province, and it is also an important meeting point between the Bohai Rim economic zone and the Beijing-Shanghai economic axis. Thus, it is one of the most important transportation hubs in eastern China. The development of Jinan's catering industry has reached a significant level in China in terms of market size, industrial structure, and richness. In this study, we selected the main urban area of Jinan as the research area (Figure 1), with an area of about 197.5 km^2^. With convenient transportation and dense catering outlets, the main urban area is the primary carrier of population and economic activities in Jinan. Therefore, it is suitable to conduct research on the street centrality and the vitality of the catering industry in Jinan.

### Data sources

The traffic road network data in the main urban area of Jinan were obtained from the National Geographic Information Public Service Platform. Combined with the research content and the actual situation, the road vector data were preprocessed. Excluding non-research objects, such as roads in parks and communities, the study area has 5,089 roads and 3,479 traffic nodes. The catering industry data came from Dianping.com and were collected from December 2020. According to the health management items in the Chinese National Standard “green hotels” (GB/T 21084-2007), we selected restaurants that can provide a safe, healthy, nutritious, and balanced diet. Selected restaurants had more than 20 dishes. We obtained 4,935 restaurant datasets, and the data for each restaurant included coordinates, merchant name, classification, score, and comment number. To exclude the influence of other factors, such as spatial location differences, on the vitality of the catering industry, we selected the intensities of various types of land, the number of bus stops, and the distance from downtown as the control variables (Table 1). Comprehensively consider the selection of influencing factors and the size of the study area, we used ArcGIS software to create a 500 × 500 m fishing net. A total of 870 grids were obtained, which were used for geographic detection and MGWR analysis.

**Table 1:** Variable descriptions.

**Variable properties**	**Variable names**	**Variable** **symbols**	**Quantitative standards**	**Variables explained**
Dependent variable	Vitality of the catering industry	Lif	KDEs of healthy restaurants based on the number of online comments	The activity level of a healthy catering industry in the region
Core explanatory variables	Closeness	Cos	Centrality value of closeness	The proximity of an area to other areas
	Betweenness	Bet	Centrality value of betweenness	The regional traffic transfer capacity
	Straightness	Str	Centrality value of straightness	The regional traffic efficiency
Control variables	Intensity of industrial land	Ind	KDEs based on floor area ratio	Land use intensity of industrial land
	Intensity of commercial land	Com	KDEs based on floor area ratio	Land use intensity of commercial land
	Intensity of public service land	Pub	KDEs based on floor area ratio	Land use intensity of public service land
	Intensity of residential land	Res	KDEs based on floor area ratio	Land use intensity of residential land
	Number of bus stops	Bus	KDEs based on number of bus stops	Accessibility to public transport
	Distance from downtown	Dis	Straight line distance from the restaurant to spring city square	Location of catering outlets in the city

### Research methods

#### Street centrality

According to the “Jinan 15-min Community Life Circle Planning” issued by the Jinan municipal government, we assume that the average travel distance of walking and driving is 1,500 m and 7,500 m, respectively when residents go out to eat. Based on the MCA model, we calculate the street centrality when the search distance threshold is 1,500 m and 7,500 m, to simulate the road network structure characteristics of walking and driving modes. Based on ArcGIS software, we selected the three indicators of closeness, betweenness, and straightness, and measured the street centrality of the main urban area of Jinan under the two travel modes. The definitions and calculation method for each indicator are described in what follows.

Closeness refers to the proximity of a node to all other nodes, which reflects the relative accessibility of the node in the transportation network. The formula is as follows (42):


(1) $$\begin{array}{l} C_{i}^{c}=\left(N-1\right)/\sum_{j=1,j\neq i}^{N}d_{ij}, \end{array}$$

where $C_{i}^{C}$ represents the closeness of node *i, N* represents the total number of nodes, and *d*_*ij*_ represents the shortest distance of the actual route from node *i* to node *j*.

According to the concept of betweenness, there is a shortest path connecting any two nodes in the transportation network, and the more shortest paths pass through a certain area, the better the betweenness of the place. The betweenness can reflect the traffic volume passing through a node—that is, the higher the betweenness value, the greater the traffic volume passing through the node. The formula is as follows (43):


(2) $$\begin{array}{l} C_{i}^{B}=\frac{1}{\left(N-1\right)\left(N-2\right)}\sum_{j=1,k=1,j\neq k\neq i}^{N}\frac{n_{jk}\left(i\right)}{n_{jk}}, \end{array}$$

where $C_{i}^{B}$ represents the betweenness of node *i, N* represents the total number of nodes, *n*_*jk*_ represents the number of shortest paths from node *j* to node *k*, and *n*_*jk*_(*i*) represents the number of paths passing through node *i* in the shortest path from node *j* to node *k*.

Straightness is an effective means to measure the traffic efficiency of a node. The smaller the ratio of the distance of the shortest path between the two nodes to the distance of the straight line (i.e., the smaller the deviation of the shortest path from the straight line), the higher the traffic efficiency between the two nodes. The formula is as follows (44):


(3) $$\begin{array}{l} C_{i}^{S}=\frac{1}{N-1}\sum_{j=1,j\neq i}^{N}\frac{d_{ij}^{Eucl}}{d_{ij}}, \end{array}$$

where $C_{i}^{S}$ represents the straightness of node *i, N* represents the total number of nodes, *d*_*jk*_ represents the shortest distance of the actual route from node *j* to node *k*, and $d_{ij}^{Eucl}$ represents the Euclidean distance from node *i* to node *j*.

#### Geographical detectors

Geographical detectors are a set of statistical methods that reveal the comprehensive interaction characteristics behind multi-factor-driven differentiation through the influence measurement of different factors based on the spatial stack and spatial collection of geographic information. We used geographic detectors for differentiation and factor detection, and we investigated the extent to which each influencing factor explained the spatial differentiation of the vitality of the catering industry. To measure the influence of each detection factor on the vitality of the catering industry, the expression is as follows (45):


(4) $$\begin{array}{l} q=1-\frac{\sum_{h=1}^{L}N_{h}\sigma_{h}^{2}}{N\sigma^{2}}, \end{array}$$

where *h* is the stratification of the impact factor; *N*_*h*_ and *N* are the number of units in the stratum *h* and the whole area, respectively; and $\sigma_{h}^{2}$ and σ^2^ are the variance of the vitality of the catering industry in the stratum *h* and the whole area, respectively. The value range of *q* is [0,1], and the smaller the value, the smaller the explanatory power of the factor *X* to the dependent variable *Y*. When *q* = 1, the factor *X* determines the spatial distribution of the dependent variable *Y*, and when *q* = 0, the factor *X* does not affect the dependent variable *Y* at all.

#### Multiscale geographically weighted regression

According to the first law of geography, the closer the distance between two things, the greater the connection. The geographically weighted regression (GWR) model shows the natural connection between items by giving higher weights to the observations in the neighborhood of the sample points. The structural equation of the classical GWR model is as follows (46, 47):


(5) $$\begin{array}{l} Y_{i}=\beta_{0}\left(u_{i},v_{i}\right)+\sum_{k}\beta_{k}\left(u_{i},v_{i}\right)X_{ik}+\varepsilon_{i}, \end{array}$$

where *Y*_*i*_ is the dependent variable at point *i*; β_0_(*u*_*i*_, *v*_*i*_) is the intercept; β_k_(*u*_*i*_, *v*_*i*_) is the value of the continuous function β(*u*_*i*_, *v*_*i*_) at point (*u*_*i*_, *v*_*i*_); *X*_*ik*_ is the value of the *k*_th_ predictor at point *i*; and ε_*i*_ is the residual.

Compared with traditional regression models, such as ordinary least squares (OLS), the classical GWR model takes into account the spatial heterogeneity of influencing factors to a certain extent by means of local regression. It uses a single kernel function and bandwidth, however, to calculate the weights, which results in the same scale characteristics for the spatial variation of all parameter estimates (48, 49). In contrast, each regression coefficient of the MGWR model is obtained based on local regression, and the bandwidth is specific, which can explain the spatial scale effect of socioeconomic phenomena (50). The linear regression equation for the MGWR is as follows (51):


(6) $$\begin{array}{l} Y_{i}=\sum_{k=1}^{q}\beta_{0}X_{ik}+\sum_{k}\beta_{bwk}\left(u_{i},v_{i}\right)X_{ik}+\varepsilon_{i}, \end{array}$$

where *bwk* represents the bandwidth used by the *k*_th_ variable regression coefficient. We used the Gaussian function method as the weight function and select the Akaike information criterion (AIC) to optimize the bandwidth.

## Results and discussions

### Spatial differentiation characteristics of the VHCI and street centrality

**Figure 2:**
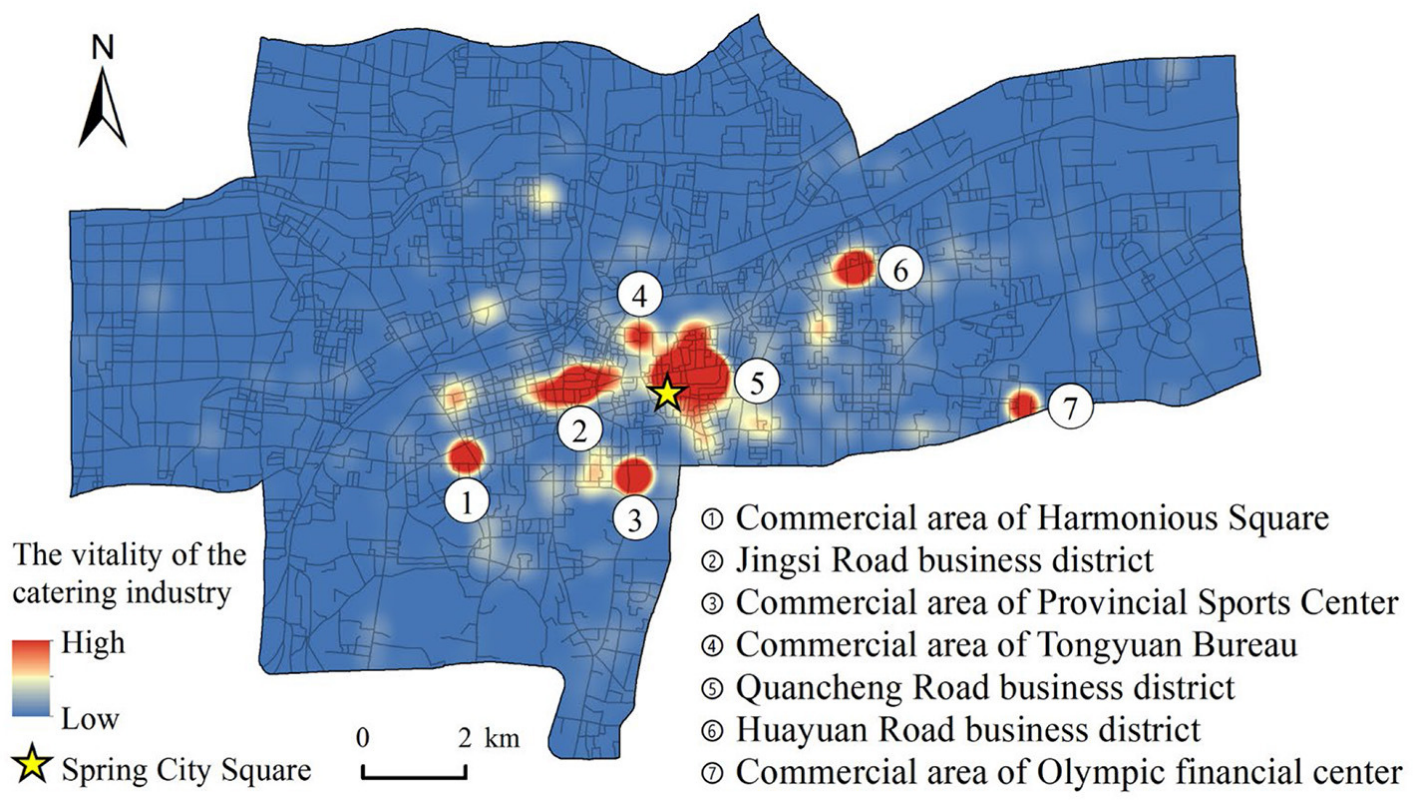
Main business districts and KDEs of the catering industry.

**Figure 3:**
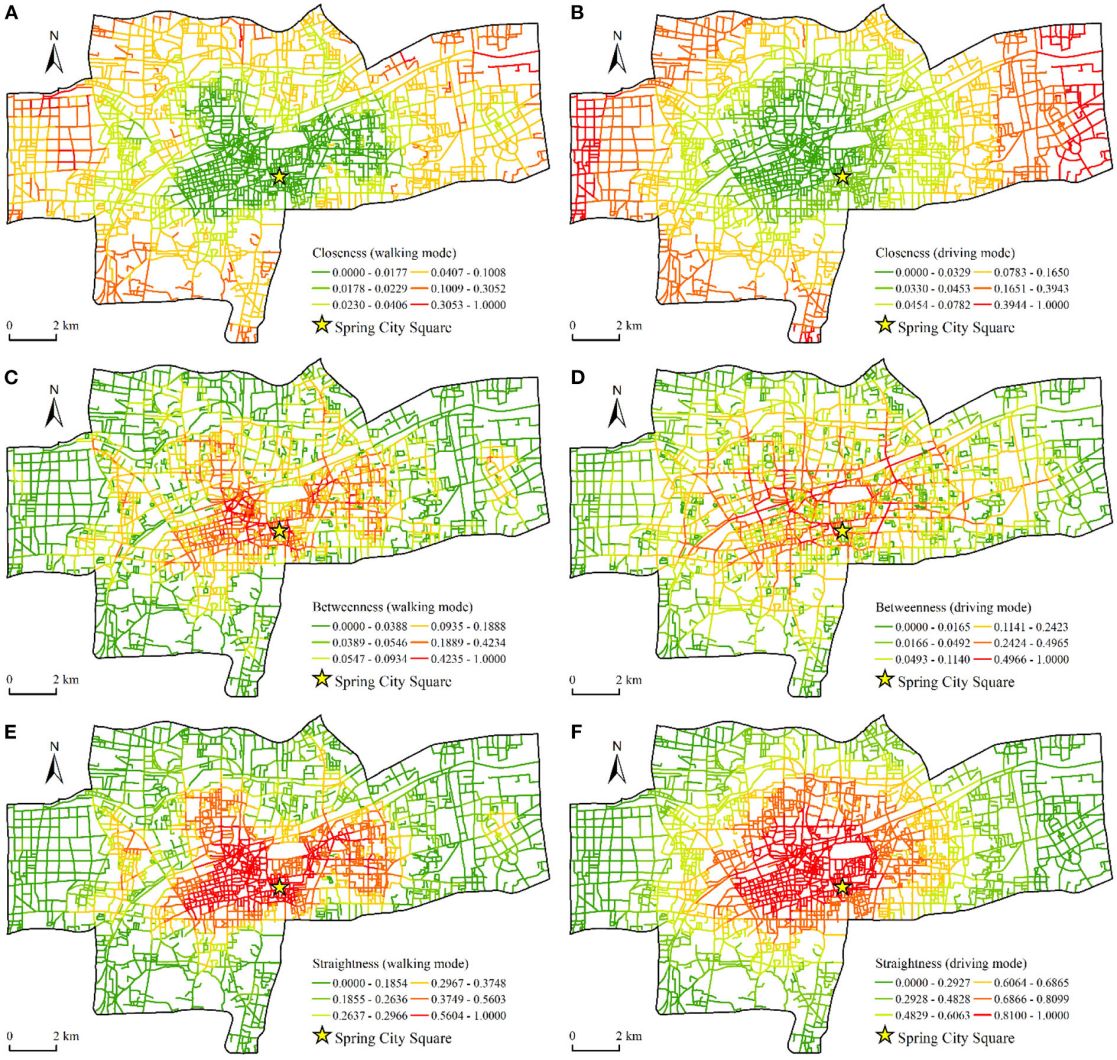
Spatial pattern of street centrality. **(A)** Closeness of walking mode. **(B)** Closeness of driving mode. **(C)** Betweenness of walking mode. **(D)** Betweenness of driving mode. **(E)** Straightness of walking mode. **(F)** Straightness of driving mode.

Taking the number of comments as the weight, we conducted the KDEs of healthy catering outlets and obtained a spatial distribution map of the vitality of the catering industry (Figure 2). Using road intersections and endpoints as nodes for street centrality analysis, we created a road network dataset in the study area. The street centrality was measured using the urban network analysis tool (Figure 3).

As shown in Figure 2, the spatial distribution of the vitality of the catering industry in Jinan presents an obvious multicenter structure with two large high-value areas. The first is the area with Quancheng Road business district as the core. This area is the center of Jinan, and it not only has famous scenic spots, such as Daming Lake and Baotu Spring, but also popular streets for snacks, such as Furong Street and Kuanhouli, which handle dense crowds and feature a dynamic catering industry. The second is the area with Jingsi Road business district as the core. This area is the traditional leisure and entertainment center of Jinan. The catering industry developed earlier in this center and the consumer market is huge. It features five smaller high-value areas, namely, the commercial area of Harmony Square, the commercial area of the Provincial Sports Center, the commercial area of Tongyuan Bureau, the Huayuan Road business district, and the commercial area of the Olympic Financial Center. In general, the vitality of the catering industry in the main urban area of Jinan is extremely uneven in spatial distribution. Areas with high vitality values are spatially clustered and are distributed near the main business district or commercial area in the central area of the city, whereas the catering vitality in other areas is relatively low.

**Figure 4:**
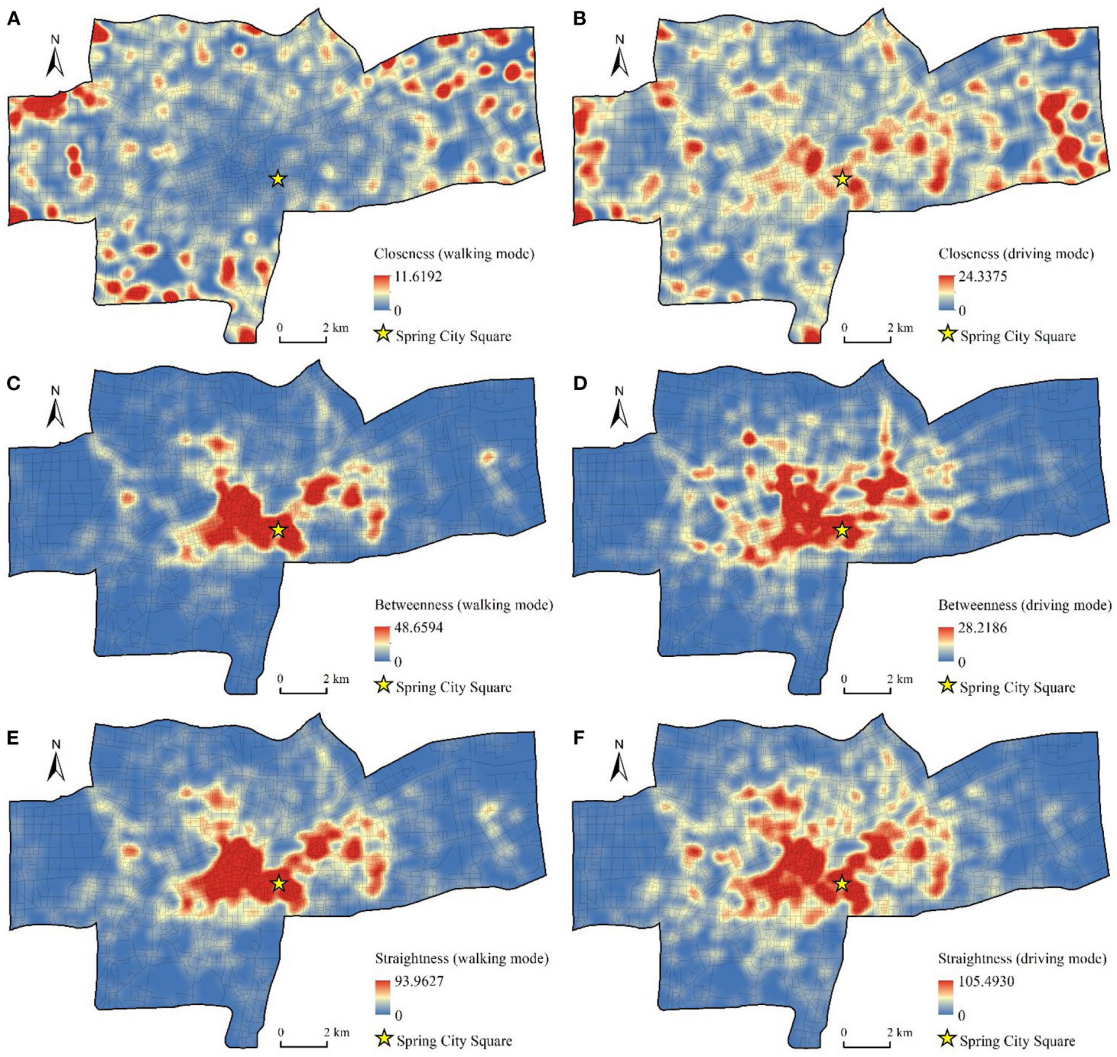
KDEs of street centrality. **(A)** Closeness of walking mode. **(B)** Closeness of driving mode. **(C)** Betweenness of walking mode. **(D)** Betweenness of driving mode. **(E)** Straightness of walking mode. **(F)** Straightness of driving mode.

In order to more clearly show the characteristics of the street centrality, we used the closeness, betweenness, and straightness as the weights to perform KDE on the nodes (Figure 4). We observed a clear spatial difference in the street centrality between the walking mode and the driving mode. From the perspective of closeness (Figures 4A,B), in the walking mode, areas with high closeness were distributed mainly at the edge of the study area and were highly discrete. This reflected the fact that at the local scale, although the number of nodes in the peripheral area was less than that in the central area, the average distance between nodes was shorter and had higher closeness. In the driving mode, the areas with high closeness gathered from the edge area to the central area. Areas with higher closeness, such as the Quancheng Road business district and the commercial area of Tongyuan Bureau, had higher values for the vitality of the catering industry.

From the perspective of betweenness (Figures 4C,D), the high-value areas of betweenness in the walking mode presented a large flaky distribution, which was concentrated in the old town in the west of Spring City Square. In the driving mode, the high-value area of betweenness expanded along the main traffic road and formed an obvious grid structure. The areas with high betweenness have played an important role in connecting and transiting between urban roads. This result indicated that the traffic nodes in these areas had large traffic flow and high commuting degree.

From the perspective of straightness (Figures 4E,F), the spatial distribution characteristics of straightness in walking mode were similar to betweenness, which showed that in the walking range of residents, the more the shortest paths between traffic nodes passed through an area, the straighter the route was in the area. In the driving mode, the straightness near the intersection of the roads has become larger, indicating that the urban traffic nodes have played a greater role in the long-distance travel of residents, and the ratio of the actual distance to the straight line between the nodes has become smaller. We found significant differences in the spatial pattern of street centrality from the perspective of these two travel modes. According to the principle of street centrality measurement, all nodes in the traffic network search for the number of nodes within a certain threshold along a road and obtain a centrality value through calculation. In the walking mode, the number of searched nodes was small, and the morphological structure of the local road network had a significant influence on the measurement of centrality; in the driving mode, the road search radius increased, which revealed a wider range of road structure characteristics.

### Influence of street centrality on the VHCI

We used Spearman's correlation coefficient to preliminarily analyze the correlation between the centrality of the road network and the vitality of the catering industry under the two modes of travel (Table 2). The results showed that the correlation between straightness and the vitality of the catering industry in the walking mode was not significant, and other street centrality indicators were significantly positively correlated with the vitality of the catering industry at the 5% significance level. Specifically, betweenness had the strongest correlation with the vitality of the catering industry in both travel modes. This result was the same as the research of Lin et al. on the retail industry in Guangzhou, China (52). The betweenness reflected the passing degree of the transportation network (53), the high-value area of betweenness was densely populated, and the vitality of the catering industry was prosperous. The correlation between straightness and the vitality of the catering industry in driving mode was greater than closeness, which means that the catering industry in areas with high traffic efficiency is more dynamic at a larger spatial scale. Among the control variables, the vitality of the catering industry was significantly negatively correlated with the intensity of industrial land and was positively correlated with the intensity of other types of land use. We found a strong positive correlation between the number of bus stops and the vitality of the catering industry, and the distance between restaurants and the downtown area had a strong negative correlation with the vitality of the catering industry.

**Table 2:** Spearman's correlation coefficient and significance test.

**Variable**	**Walking mode**			**Driving mode**			**Ind**	**Res**	**Pub**	**Com**	**Bus**	**Dis**
	**Clo**	**Bet**	**Str**	**Clo**	**Bet**	**Str**						
Lif	0.106^**^	0.663^**^	−0.028	0.436^**^	0.667^**^	0.658^**^	−0.151^**^	0.724^**^	0.625^**^	0.609^**^	0.738^**^	−0.640^**^

** means the confidence level is 95%.

**Figure 5:**
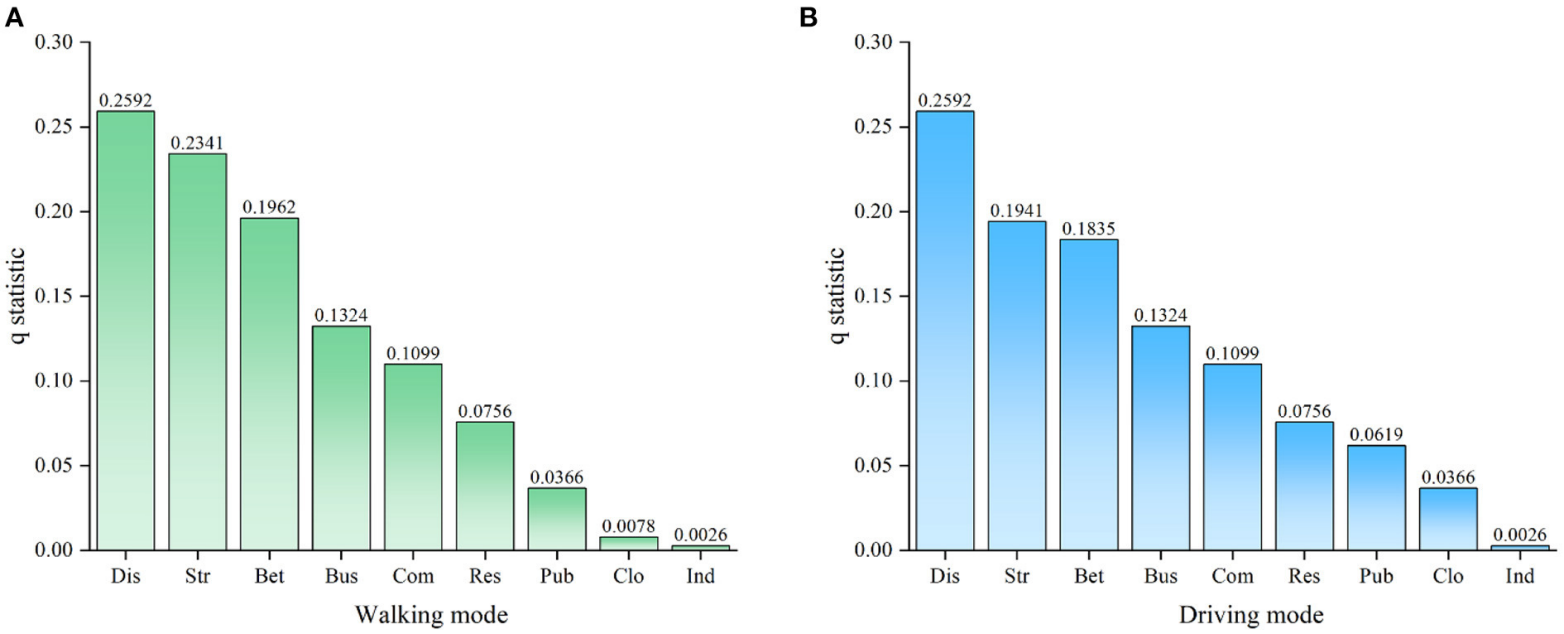
Influence of street centrality and control variables on the vitality of the catering industry. **(A)** q statistics of walking mode. **(B)** q statistics of driving mode.

Spearman's correlation coefficient represents the linear relationship between variables, but it does not reflect the impact of street centrality on the vitality of the catering industry. Therefore, we used geographic detectors to further analyze the extent to which street centrality explains the spatial differentiation of the vitality of the catering industry (Figure 5). The q statistic of the geographical detection showed the following: In the walking mode, Distance from downtown (0.2592) > Straightness (0.1941) > Betweenness (0.1835) > Number of bus stops (0.1324) > Intensity of commercial land (0.1099) > Intensity of residential land (0.0756) > Closeness (0.0619) > Intensity of public service land (0.0366) > Intensity of industrial land (0.0026). In the driving mode, Distance from downtown (0.2592) > Straightness (0.2341) > Betweenness (0.1962) > Number of bus stops (0.1324) > Intensity of commercial land (0.1099) > Intensity of residential land (0.0756) > Intensity of public service land (0.0366) > Closeness (0.0078) > Intensity of industrial land (0.0026).

The results of geographical detectors showed that the impact of the street centrality index on the vitality of healthy catering industry under the two travel modes was significantly different. After considering the spatial effects of independent and dependent variables, straightness had the greatest impact on the vitality of the catering industry, followed by betweenness and closeness. This meant that the degree of deviation between the shortest path and the straight path between nodes in the area had the greatest impact on the vitality of the catering industry, the number of the shortest paths between nodes had a secondary impact on the vitality of the catering industry, and proximity had the least impact on the vitality of the catering industry. This result was different from the conclusion of Scopa et al. on the relationship between the distribution of commercial facilities and street centrality in Buenos Aires (54). The reason for this is that the street centrality index plays a different role in the road network structure of different cities. Catering outlets are greatly affected by the traffic network. To obtain greater benefits and meet the needs of more consumers, these outlets tend to be distributed in areas with superior traffic conditions (55). At the same time, the goal-oriented travel habits of urban residents have made the regional catering industry with high traffic efficiency and strong transit ability more dynamic (56). The straightness represents traffic efficiency, and the betweenness represents transit capacity. These two factors have had a significant impact on the vitality of the catering industry in the driving mode, which has demonstrated that the road network plays a greater role in a wider range of spatial scales (57). Road sections with strong transfer capacity often have large traffic flow and are prone to traffic congestion. At the same time, residents are more inclined to choose straight roads with low time cost and high safety (58). Therefore, the impact of betweenness on the vitality of the catering industry was less significant than that of straightness. The difference in the impact of betweenness and straightness on the vitality of the catering industry was more obvious in the driving mode because traffic congestion had a greater impact on driving than on walking. The influence of closeness on the vitality of the catering industry in the walking mode was higher than that in driving mode. When residents drove out of the urban center, the attractiveness of the neighboring areas was weaker because of the more convenient travel. Among the control variables, Distance from downtown, Number of bus stops, and Intensity of commercial land had the greatest impact on the vitality of the catering industry. These results showed that the distance between the restaurants and the downtown area, the convenience of public transportation, and the concentration of business have greatly affected the spatial distribution of the vitality of the catering industry.

### Spatial heterogeneity analysis

The global Moran's I test is used to test whether there is a non-equilibrium spatial distribution of each variable (Table 3). The results showed that both the dependent variable and the independent variable had a positive spatial correlation. Intensity of industrial land, Intensity of residential land, and Number of bus stops passed the significance test at the 5% level, and other factors passed the significance test at the 1% level. This result indicated that both the core variable and the control variable had spatial clustering between similar values, which was suitable for local regression analysis. We conducted the MGWR analysis using MGWR 4.0 software, selected the Gaussian fixed kernel as the kernel type, and selected the AIC_C_ method as the model bandwidth. We selected the AIC_C_ and adjusted *R*^2^ to compare the fitting results of OLS, GWR, and MGWR (Table 4). The MGWR model had the smallest AIC_C_ and the largest adjusted *R*^2^, and therefore, the MGWR model had a best fitting effect. The statistical description of the coefficients of each variable of MGWR is shown in Table 5.

**Table 3:** Spatial correlation test results of variables.

**Variable**	**Walking mode**			**Driving mode**			**Ind**	**Res**	**Pub**	**Com**	**Bus**	**Dis**
	**Clo**	**Bet**	**Str**	**Clo**	**Bet**	**Str**						
Moran's I	0.949^***^	0.345^***^	0.848^***^	0.692^***^	0.530^***^	0.856^***^	0.849^***^	0.455^**^	0.696^**^	0.631^***^	0.626^***^	0.676^**^

** means the confidence level is 95%,

and

*** means the confidence level is 99%.

**Table 4:** Comparison of goodness of fit of OLS, GWR, and MGWR models.

	**OLS**		**GWR**		**MGWR**	
	**AIC_C_**	**Adjusted *R*^2^**	**AIC_C_**	**Adjusted *R*^2^**	**AIC_C_**	**Adjusted *R*^2^**
Walking mode	2255.598	0.227	1921.585	0.608	1641.858	0.691
Driving mode	2284.233	0.201	1977.065	0.543	1735.088	0.651

**Table 5:** Statistical description of the coefficients of each variable in MGWR.

**Walking mode**	**Mean**	**STD**	**Min**	**Median**	**Max**	**Driving mode**	**Mean**	**STD**	**Min**	**Median**	**Max**
Clo	0.145	0.161	−0.005	0.083	0.722	Clo	0.446	0.373	0.002	0.344	1.949
Bet	0.382	0.268	−0.175	0.304	1.404	Bet	0.102	0.245	−0.579	0.109	1.150
Str	−0.577	0.288	−1.288	−0.563	0.180	Str	−0.677	0.309	−1.574	−0.732	0.042
Ind	0.012	1.041	−7.016	−0.084	4.707	Ind	−0.012	0.001	−0.013	−0.012	−0.011
Com	0.253	0.132	0.020	0.255	0.471	Com	0.212	0.130	−0.013	0.193	0.446
Pub	−0.092	0.054	−0.252	−0.083	−0.008	Pub	−0.108	0.088	−0.395	−0.081	−0.011
Res	−0.079	0.002	−0.082	−0.079	−0.077	Res	−0.065	0.002	−0.067	−0.064	−0.063
Bus	0.036	0.003	0.030	0.037	0.040	Bus	0.023	0.004	0.015	0.025	0.029
Dis	−0.961	0.915	−2.787	−1.328	0.151	Dis	−1.099	0.896	−3.060	−1.453	0.032

#### Walking mode

**Figure 6:**
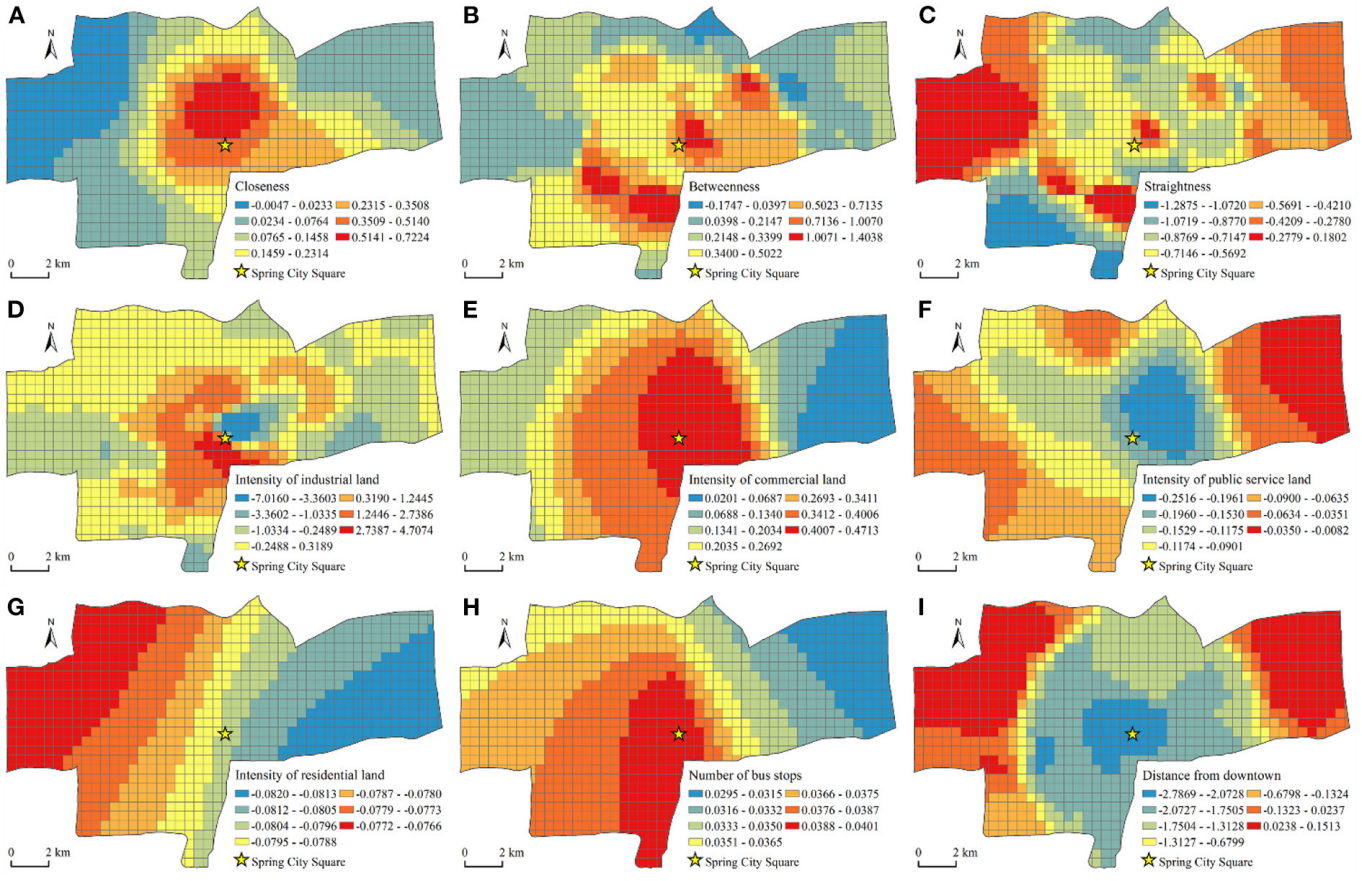
Spatial pattern of regional coefficients of MGWR variables in the walking mode. **(A)** Closeness. **(B)** Betweenness. **(C)** Straightness. **(D)** Intensity of industrial land. **(E)** Intensity of commercial land. **(F)** Intensity of public service land. **(G)** Intensity of residential land. **(H)** Number of bus stops. **(I)** Distance from downtown.

According to the results in Table 5, the closeness coefficient was less discrete in the walking mode. This result explained that the influence of closeness on the vitality of the catering industry varied slightly in different locations. As shown in Figure 6A, the spatial distribution of the closeness coefficient was in the “core-periphery” mode, with the northern area of Spring City Square as the core, and gradually decreased to the periphery. The closeness coefficient in the western area of the main urban area was the smallest. The closeness coefficient for the high-value areas was concentrated in the city center, because residents in this area have a tense life rhythm. Therefore, they pay more attention to the accessibility of a destination to save traffic time. The degree of dispersion of the betweenness coefficient was relatively high, which showed that the influence of betweenness on the vitality of the catering industry varied greatly in different locations. Figure 6B shows that the spatial pattern of the betweenness coefficient had a multicenter structure. The high-value areas were mostly business gathering areas or tourist scenic areas, and many business people and tourists came from other places. The directness coefficient had the highest degree of dispersion, which indicated that the directness had the greatest impact on the vitality of the catering industry in different locations. Figure 6C shows that the spatial pattern of the straightness coefficients also presented a multicenter pattern. The regression coefficient in the western part of the main urban area generally was higher. These areas are located far from the downtown, and the catering outlets have to attract a wider range of consumers. Because residents pay more attention to the traffic efficiency of the destination when they travel long distances, straightness has a greater impact on the vitality of the catering industry (57). The distribution of the remaining four high-value areas basically coincided with the betweenness coefficient for the high-value areas. This result showed that betweenness and straightness, as important indicators to measure the convenience of transportation, both had an important impact on consumers' choice of dining places. The straightness coefficients in the north and southwest of the main urban area were negative. The vitality of the catering industry in this area may be greatly affected by other factors. Among the control variables, the influence of the intensity of industrial land on the vitality of the catering industry had the greatest difference in the different locations. From the perspective of the spatial distribution of the regression coefficients, the regression coefficients for the main urban areas where the distribution of industrial land is concentrated were mainly negative, which indicated that the industrial land in this area would have a certain negative impact on the vitality of the catering industry. The regression coefficient in the central area of the city was positive, because the industrial production process in this area is more environmentally friendly and the industrial land area is small, which had no obvious negative impact on the vitality of the catering industry.

#### Driving mode

**Figure 7:**
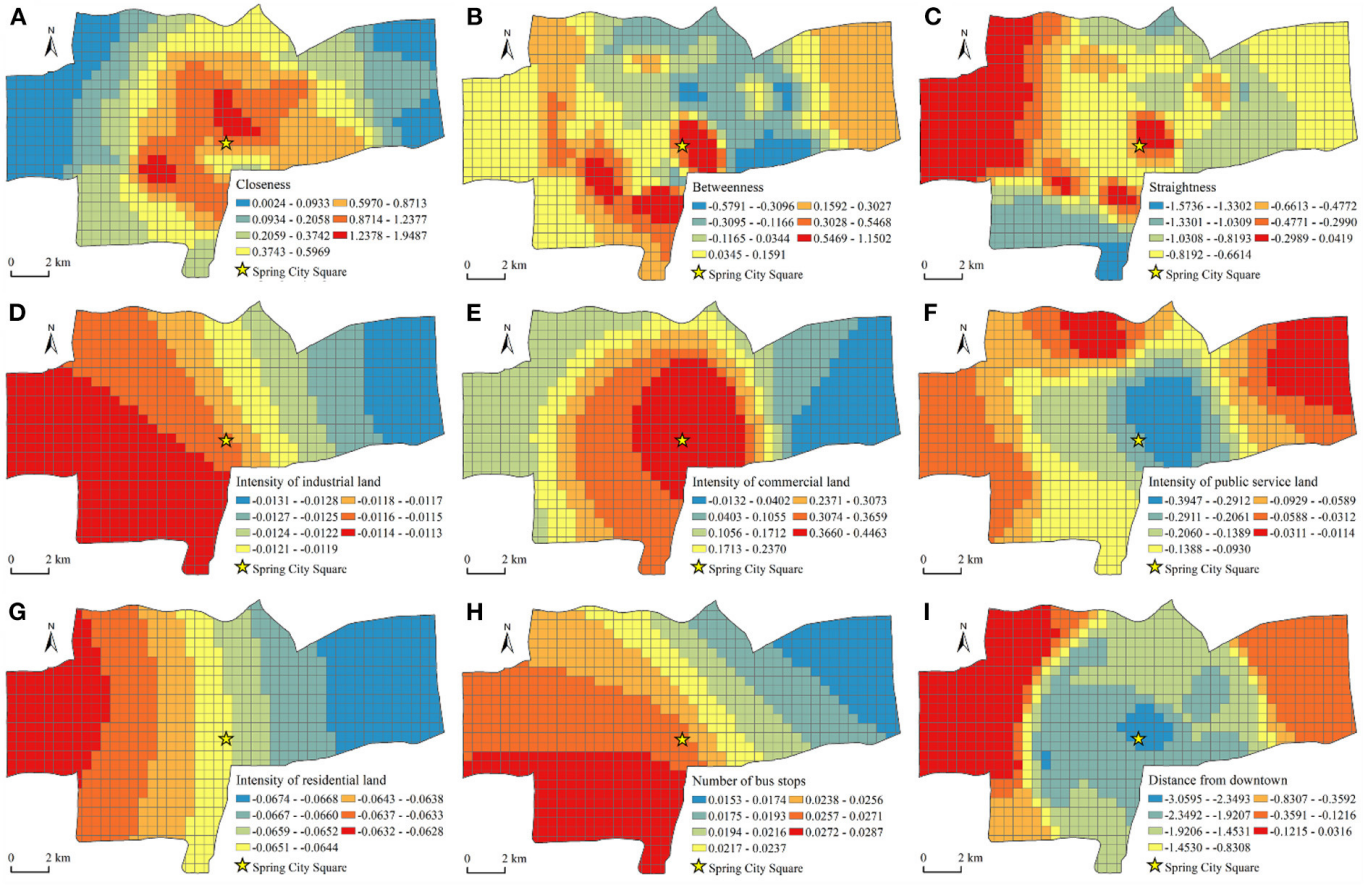
Spatial pattern of expression coefficients of MGWR variables in the driving mode. **(A)** Closeness. **(B)** Betweenness. **(C)** Straightness. **(D)** Intensity of industrial land. **(E)** Intensity of commercial land. **(F)** Intensity of public service land. **(G)** Intensity of residential land. **(H)** Number of bus stops. **(I)** Distance from downtown.

According to the results in Table 5, the spatial difference of the impact of street centrality on the vitality of the catering industry was greater in the driving mode than in the walking mode. In the regression coefficient of the street centrality index, the closeness coefficient had the highest degree of dispersion. Under the driving mode, the influence of closeness in different locations on the vitality of the catering industry had the most obvious difference. As shown in Figure 7A, compared with the walking mode, the distribution of the closeness coefficient exhibited a ring-layer structure that gradually decreased from the center to the periphery, but the high-value area extended from the center to the southwest. The two core areas were the Quancheng Road business district and commercial area of Harmonious Square. This indicated that the increase in travel distance changed the local characteristics of the influence of closeness on the vitality of the catering industry. As shown in Figure 7B, the degree of dispersion of the betweenness coefficient was small, which indicated that the influence of betweenness on the vitality of the catering industry was not very different in different regions. As an important indicator to measure the convenience of transportation, betweenness had an important influence on the vitality of the catering industry in most areas. The commercial area of Harmonious Square, the commercial area of the Provincial Sports Center, and the Quancheng Road business district together constitute the high-value areas of the betweenness coefficient. Compared with the walking mode, the betweenness coefficient of the urban central area became smaller, and the edge coefficient of the study area became larger. The public transportation in the central area is developed, and residents can travel by bus or subway. The influence of betweenness on the vitality of the catering industry has been weakened. Conversely, public transportation is inconvenient in the fringe areas and the proportion of private car trips is relatively large, and thus areas with high betweenness have greater transportation advantages. The degree of dispersion of the straightness coefficient was higher than betweenness and was secondary to closeness. As shown in Figure 7C, the straightness coefficient in the driving mode was also in a multicore mode, and the distribution characteristics of the high-value areas were similar to those in the walking mode. Judging from the absolute value of the coefficient, the impact of straightness on the vitality of the catering industry west of the main city was particularly prominent. The road network in this area is relatively sparse, and choosing restaurants with high straightness saves travel time. Among the control variables, the distance from downtown was the biggest factor affecting the spatial difference of the vitality of the catering industry. The regression coefficient gradually increased from Spring City Square to the periphery, which indicated that the promotion effect of the city center location on the vitality of the catering industry weakened with an increase in distance. The absolute values of the coefficients on the western and eastern edges of the main urban area were relatively small. For restaurants, the location advantage of the downtown existed only within a certain distance threshold. After the distance threshold was exceeded, the attractiveness of the central location decreased rapidly.

## Conclusion and planning implications

### Major findings

By analyzing the relationship between street centrality and the vitality of healthy catering industry, the following conclusions can be drawn:

First, the vitality of the catering industry in the main urban area of Jinan presented a multicore spatial distribution model. We identified two cores, the Quancheng Road business district and Jingsi Road business district, and five subcenters, but most of these areas had low or no vitality. The high value areas of vitality coincided with the main business districts of the city in terms of spatial distribution. We found obvious differences in the spatial pattern of street centrality in the walking and driving modes. As the measurement scale of street centrality became larger, a wider range of traffic road network characteristics was revealed. The high-value area of closeness tended toward the downtown area from the edge of the study area, the betweenness was distributed from sheet to grid, and the multicenter structure of straightness was more protruded.

Second, street centrality affected the vitality of the catering industry by affecting regional traffic flow and traffic efficiency. In general, straightness had the greatest impact on the vitality of the catering industry, followed by betweenness and closeness. Changes in residents' travel patterns led to differences in the perception of the road network structure, which in turn affected destination choices. On the large-scale spatial scale, betweenness and straightness had a greater impact on the vitality of the catering industry, and closeness was more obvious on the small spatial scale.

Third, residents' different living conditions, travel habits, and travel needs also affected the choice of restaurants. Street centrality of the two travel modes had different influences on the vitality of the catering industry in different locations. In the walking mode, the straightness had the strongest spatial heterogeneity, the betweenness was the second, and the closeness was the weakest. In the driving mode, the closeness had the strongest spatial heterogeneity, the straightness was the second strongest, and the betweenness was the weakest.

## Planning implications

The findings of this study have policy implications for the key role of road network structure in the healthy living environment of urban residents. First, the overall positive spatial connection between street centrality and VHCI shows that the spatial features of human activities, such as geographic location, are necessary for scientific urban planning (57). Authorities should make use of the location advantage of the road network, the construction of the urban business districts and the planning of the living circle to guide the design of a healthy urban environment, provide a beneficial location for restaurants that provide healthy food, and actively guide the green and healthy development of the catering industry. Second, urban designers and planners should realize that the advantages of a street network configuration cannot be determined on a single scale. On the contrary, various travel modes should be considered, taking into account the healthy dietary needs of different groups of people, and promoting the improvement of urban quality (59). The results of our study indicate key factors that need to be considered in the construction of a healthy urban environment, and provide a useful reference for urban space optimization. However, residents' travel habits and dietary preferences vary from region to region, so further research is needed to determine the specific impact of different regions. With more evidence, it is easier to extend the findings of one region to others and provide information for healthy urban planning in China and other countries (60).

### Limitations

Influenced by data sources, analysis models, and methods, this study will be further improved in the following two ways: First, the influence of road grade, width, and other factors was not considered when calculating the street centrality, which may have caused the deviation between the calculation results and the actual situation. Second, because of data acquisition limitations, this study analyzed the impact of street centrality on the vitality of the catering industry based on cross-sectional data and failed to reflect the dynamic characteristics of the vitality of the catering industry and its influencing factors. In the future, data for different types of cities in different years will be collected, and a spatiotemporal dynamic analysis of street centrality and the vitality of healthy catering will be conducted.

## Data availability statement

The original contributions presented in the study are included in the article/supplementary material, further inquiries can be directed to the corresponding author.

## Author contributions

Conceptualization, formal analysis, and methodology: YC and YH. Data curation and software: YH. Funding acquisition and project administration: GY. Supervision and writing—review and editing: GY and YH. Writing—original draft: YC. All authors contributed to the article and approved the submitted version.

## Funding

This research was funded by the National Natural Science Foundation of China (41601156).

## Conflict of interest

The authors declare that the research was conducted in the absence of any commercial or financial relationships that could be construed as a potential conflict of interest.

## Publisher's note

All claims expressed in this article are solely those of the authors and do not necessarily represent those of their affiliated organizations, or those of the publisher, the editors and the reviewers. Any product that may be evaluated in this article, or claim that may be made by its manufacturer, is not guaranteed or endorsed by the publisher.
